# The Ontogeny of Gap Crossing Behaviour in Bornean Orangutans (*Pongo pygmaeus wurmbii*)

**DOI:** 10.1371/journal.pone.0130291

**Published:** 2015-07-08

**Authors:** Jackie Chappell, Abigail C. Phillips, Maria A. van Noordwijk, Tatang Mitra Setia, Susannah K. S. Thorpe

**Affiliations:** 1 School of Biosciences, University of Birmingham, Birmingham, United Kingdom; 2 Anthropological Institute and Museum, University of Zurich, Zurich, Switzerland; 3 Facultas Biologi, Universitas Nasional, Jakarta, Indonesia; University of Florence, ITALY

## Abstract

For orangutans, the largest predominantly arboreal primates, discontinuous canopy presents a particular challenge. The shortest gaps between trees lie between thin peripheral branches, which offer the least stability to large animals. The affordances of the forest canopy experienced by orangutans of different ages however, must vary substantially as adult males are an order of magnitude larger in size than infants during the early stages of locomotor independence. Orangutans have developed a diverse range of locomotor behaviour to cross gaps between trees, which vary in their physical and cognitive demands. The aims of this study were to examine the ontogeny of orangutan gap crossing behaviours and to determine which factors influence the distance orangutans crossed. A non-invasive photographic technique was used to quantify forearm length as a measure of body size. We also recorded locomotor behaviour, support use and the distance crossed between trees. Our results suggest that gap crossing varies with both physical and cognitive development. More complex locomotor behaviours, which utilized compliant trunks and lianas, were used to cross the largest gaps, but these peaked in frequency much earlier than expected, between the ages of 4 and 5 years old, which probably reflects play behaviour to perfect locomotor techniques. Smaller individuals also crossed disproportionately large gaps relative to their size, by using support deformation. Our results suggest that orangutans acquire the full repertoire of gap crossing techniques, including the more cognitively demanding ones, before weaning, but adjust the frequency of the use of these techniques to their increasing body size.

## Introduction

The ability to move from one tree to the next and cross small gaps in the forest canopy is a key factor in minimising path length for arboreal species [1] and therefore for optimising the daily energetic cost of locomotion. Orangutans are the largest habitually arboreal mammal and, despite their diverse repertoire of positional behaviour [2–3], transferring between trees (hereafter gap crossing) is likely to be particularly challenging for them because the narrowest gaps between tree crowns are found between thin compliant branches [4], which should deflect considerably under their large weight.

Most studies have found that the oscillations of compliant terminal branches caused by animals moving along them increase the energetic costs of arboreal locomotion e.g. [5–6]. Some species however, including orangutans, can use compliant branches and lianas to their advantage during gap crossing [7–8]. In such interactions, orangutans manipulate compliant supports in 2 ways: appendicular deformation and mass deformation [7]. Appendicular deformation is generally used when the branches of an adjacent tree are within reach, and orangutans cross by pulling thin compliant branches towards their body until a more stable branch can be reached to transfer their weight onto. They use this behaviour during both orthograde (upright-torso) transfer and pronograde (horizontal-torso) bridging behaviour [3, 9]. Mass deformation is where a support is intentionally deformed using body mass [7]. It is used when crossing larger gaps in the canopy and might either be with the animal in a static position with the support simply deflecting under their mass, or when orangutans actively sway supports with increasing amplitude until a support on the opposite side of the gap can be reached, such as in tree sway [10–13]. Tree sway has been found to be less than half as energetically costly as jumping (which also carries a increased risk of falling) and an order of magnitude less costly than crossing terrestrially for a similar sized gap [8].

The affordances of the forest canopy experienced by orangutans of different age categories however, vary substantially since adult male Bornean orangutans may weigh up to 90kg, which is an order of magnitude greater than infants during their first forays into independent locomotion. Whereas adult orangutans may use their large body weight to facilitate gap crossing by exploiting compliant supports (as in tree sway), immature orangutans may be too light to deform the supports sufficiently to cross the same gap and may have to rely on their mother to assist their crossing or take an alternative route. Adults also have longer, larger and stronger hands and feet, which extends their reach and allows them to grasp multiple branches and lianas with each limb and distribute their body mass across multiple peripheral branches. Conversely however, smaller body mass may make crossing easier because smaller animals are less likely to break thin branches at the periphery of trees, which allows them to take more continuous travel routes [12].

Despite these functional differences, previous studies have found a limited influence of age-sex category on positional behaviour and support use [3,13–15]. Thorpe and Crompton [13] proposed two reasons for the congruence in positional behaviour across the age-sex classes. Firstly, orangutans of all classes were found to follow the same travel routes or ‘arboreal pathways’ [13] and, as they found a strong association between support type and positional behaviour, this may have minimized differences. Secondly, parous adult females were found to exhibit particularly cautious locomotor behaviour, preferring large stable supports similar to those used by the much heavier males [13]. However none of these studies collected data from dependent immature individuals (i.e. those <7 years old) [2–3, 11, 13–17], which may have masked the extent of the impact of body mass on locomotor behaviour. Although orangutans in this age range are dependent on their mother for transport to varying degrees (orangutan mothers carry their offspring for most longer distance travel during the first 2–3 years and still assist in crossings up to 6–7 years of age), independent locomotor behaviour is also developing [18–19].

Gap crossing skills have been shown to be cognitively challenging for young orangutans to learn [20–21], which indicates that gap crossing may be an important constraint on the development of independent locomotor abilities in immature orangutans. Orangutans must be able to perceive the affordances of supports to utilize them successfully and they discover these through play and exploratory behaviour [22], much like human infants [23–25], or by observing their mothers when they are carried or assisted across gaps. Orangutan mothers further encourage independent behaviour in their infants by systematically reducing the assistance they provide as offspring gain competence [20]. While appendicular deformation of compliant supports does not appear to require much experience, certain forms of mass deformation are likely to require more advanced cognitive abilities and/or more experience [21]. For example during tree sway orangutans often deform supports away from their intended travel direction in order to increase the magnitude of the sway. Chevalier-Skolnikoff et al. [21] suggested that this behaviour indicates that orangutans cross gaps by forming mental representations of them prior to crossing, which they consider indicative of the most cognitively complex stage of Piaget’s sensorimotor intelligence series, *insight*. Since the extent to which a substrate deforms depends on the body mass of the animal, orangutans must also adapt their locomotor behaviour as they grow. As young orangutans differ in both body mass and cognitive development, age-related differences in locomotor behaviour should be particularly pronounced in immature orangutans.

The aim of the present study was to investigate the development of gap crossing behaviour in orangutans ranging from infant to adult. We achieved our aim by testing the hypotheses that: 1) the exploitation of compliance during gap crossing will increase as body size increases. In particular, mass deformation will be more common in the gap crossing locomotion of heavier orangutans; 2) the size of the gap crossed will increase as body size increases; 3) the skills required to cross a gap using mass deformation will develop later than appendicular deformation skills as mass deformation techniques are both cognitively and physically challenging.

## Methods

### Study site and subjects

This study was carried out at Tuanan research area (2°09’S, 114°26’E) within the Mawas Reserve, Central Kalimantan, Indonesia. Appropriate permits and approvals were obtained for this study from RISTEK, the Director General Departemen Kehutanan (PHKA), Departamen Dalam Negri, the local government in Central Kalimantan, BKSDA Palangkaraya, the Bornean Orangutan Survival Foundation (BOSF and MAWAS).

The study area consists of approximately 725 ha of lowland peat swamp forest with an orangutan density of 4.25/km^2^ [26]. The area was subject to selective logging in the early-mid 1990s, prior to the onset of continuous research in 2003. The subjects were 12 wild habituated Bornean orangutans (*Pongo pygmaeus wurmbii*) ranging from infant to adult (Table 1). As this was a cross-sectional study of locomotor development, subjects of different ages, and therefore body sizes, were selected to represent different stages of development. Immature and adult subjects of both sexes were sampled.

**Table 1:** Subject information.

Subject	Sex	Age at observation (years)^1^	Forearm length (cm)	Focal days	Num. observed crossings	Mean AGC (m)	AGC^2^ range (m)
Mawas	female	**1**	12.8	10	90	0.06	0–1
Kino	male	**3**	17.4	10	613	0.31	0–4
Jip	Male	**4**	20.7	10	819	0.79	0–3
Deri	Male	**5**	23.5	7	545	1.00	0–5
Jerry	Male	**6**	27.3	10	1172	1.07	0–5
Streisel	Female	7	29.9	9	820	1.02	0–6
Milo	Female	9	31.3	7	974	1.17	0–6
Kondor	Female	11	33.2	10	1405	1.17	0–6
Juni	adult female	15–20	35.4	5	739	1.34	0–5
Kerry	adult female	>25	38.0	5	463	1.57	0–5
Gismo	unflanged male male	>20	45.6	4	678	1.57	0–5
Preman	flanged male	>25	50.9	3	334	1.35	0–5

^**1**^Age (year and month of birth) of immatures < 7 yr old were known (depicted in bold), for those 7 years and older ages were estimated on age of their offspring or morphological characteristics (see also [27]).

^2^AGC is actual gap crossed (distance from take off point or last point before support deformation to landing point).

### Measurement technique

To quantify the body size of each individual a non-invasive laser photography technique [28] was used whereby 3 parallel green lasers were attached to a camera (Canon EOS 400D) using a specifically designed aluminium frame. The lasers were held in the frame 4cm apart and provided visible marks on the photograph that formed a scale bar. The laser beams were regularly calibrated to ensure they were exactly 4 cm apart. The technique was validated using laser projections on tree branches (n = 30) where precise measurements were taken manually by an experienced tree climber. The mean error was less than 1% indicating that measurements obtained from photographs using parallel lasers were accurate [29].

As orangutans have a high intermembral index their forelimbs show greater variation in length than their hindlimbs. The forelimb is also functionally relevant in determining how far the animal can reach across a gap for a support. In practice it was easier to measure the forearm than the upper arm in photographs because locating the shoulder joint was more difficult than locating the wrist. Forearm length, measured from the prominent olecranon process of the ulna to the radio-carpal joint at the wrist, was therefore chosen to represent functional body size in this study.

The lasers were used to measure the subjects’ forearms, which were photographed when they were perpendicular to the field of view of the camera to avoid errors associated with foreshortening. Photographs of subjects were taken during the same observation period as focal sampling. Some modification was made to the original method as Rothman [28] measured monkey tails which reliably hang vertically but orangutans limbs rarely do that. Thus, multiple photographs of each subject were taken when the forelimbs appeared perpendicular to the field of view of the camera, and later measured using Image J version 1.43 (Bethesda, MD). The largest value was taken to be the closest to the true length because foreshortening can only cause underestimation.

### Data collection

The study was carried out from June to November 2009 and January to July 2010. Continuous observations of focal subjects were carried out from when the subject awoke from their nest in the morning to when they rested in their evening nest, typically from 5 am to 5 pm. Subjects were followed for a maximum of ten sequential days per month to limit continuous exposure to observers. However, to avoid bias associated with temporarily abundant food sources [13], effort was also made to study each individual on more than one occasion. Where it was possible to relocate the individuals, the interval between observation periods was less than 6 months, to avoid blurring the boundaries between age groups.

In our definition of an independent gap crossing event we excluded events where the infant was carried by its mother or where the movement of the support was entirely instigated by the mother, but we included crossings where, for example, the mother made a bridge and the infant crossed between trees higher up by pulling in adjacent branches independently. Gap crossing locomotion was initially recorded following the standard classification system for primate positional behaviour [3,9], which classifies locomotor behaviour according to body orientation, weight bearing limbs and whether weight is borne in suspension or compression (see [30] for full list of locomotor behaviours exhibited in this study). However to address the questions in this study gap crossing locomotion was reclassified according to the animal’s interaction with compliant supports. Gap crossing behaviours therefore included: appendicular deformation, where the orangutan crossed by pulling thin peripheral branches from the destination tree towards their body until a more stable branch could be reached to transfer their weight onto; ride, in which the animal used its mass to deform a support in the direction of travel; and sway in which mass deformation was used to oscillate a compliant support back and forth with increasing amplitude until the destination tree could be reached. In practice orangutans sometimes combined ride or sway with appendicular deformation, when for example they used mass deformation to cross the majority of the gap and then appendicular deformation to transfer into the next tree, and these were classed accordingly. Locomotion that did not utilize a compliant support was termed non-compliance utilising (NCU).

**Fig 1:**
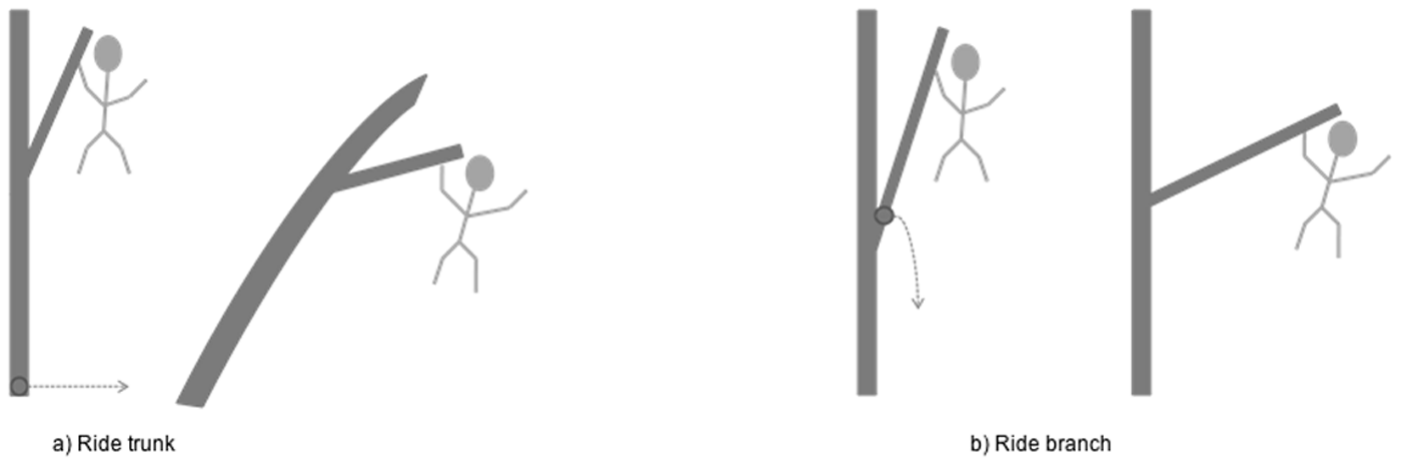
Illustrations to show the definition of the take-off support. The take-off support during the gap crossing behaviour ride was recorded as the support that deformed rather than the support the animal was holding on to. A) ride on trunk and B) ride on branch.

Every time focal orangutans were observed crossing between trees locomotor behaviour, support use and an estimate of the size of the gap crossed were recorded using a digital voice recorder to enable the observer (ACP) to watch and record simultaneously. Three types of support were distinguished: trunks (the primary members of trees), branches (all other tree elements including twigs and foliage), and lianas (vines with woody stems). This study recorded the support that was used to make the crossing. For modes that utilised support compliance the support that was deformed to make the crossing was recorded (see distinction in Fig 1a and 1b). For all other modes of locomotion, the last support that the subject made contact with on the take-off tree was recorded. Self-training in estimating horizontal distances in the forest was carried out extensively before the study and at monthly intervals during the study to ensure estimations were accurate. This was achieved by estimating horizontal distances at a range of heights in the canopy that could be subsequently quantified accurately at ground level.

**Fig 2:**
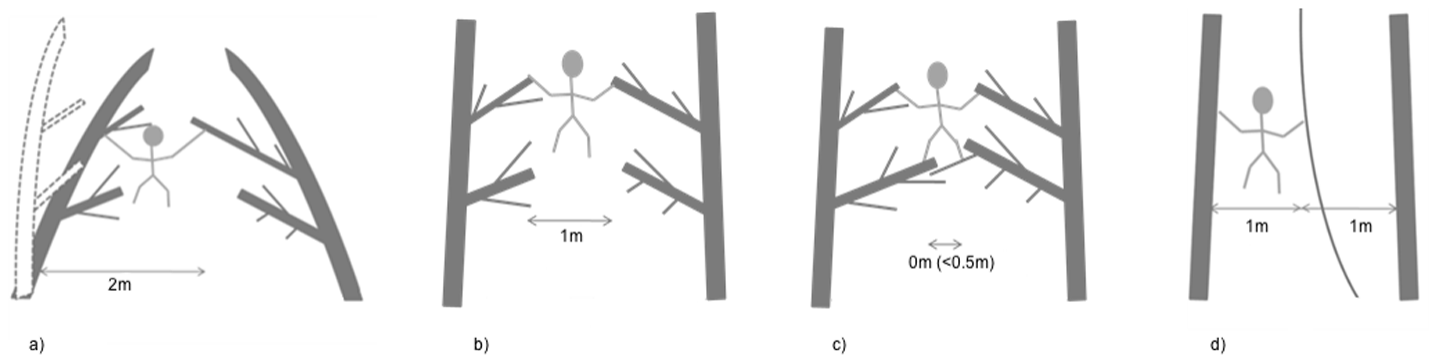
Illustrations of how the actual gap crossed by orangutans was estimated during different types of gap crossing. When the gap was crossed using mass deformation, the take-off point was the last location of the orangutan prior to support deformation (Fig 2a). For all other types of behaviour the take-off point was the last part of the take-off tree that the orangutan made contact with (Fig 2b). If this distance was estimated to be less than 0.5m the AGC was recorded as zero (Fig 2c). AGC was estimated to the nearest whole metre. In cases where a subject crossed a gap to get to a liana and then used the liana to get to the next tree, details of both crossings were recorded (Fig 2d).

The deflection of terminal branches under the weight of arboreal animals during gap crossing has resulted in the development of several methods to estimate the size of the gap crossed. Gebo [31] estimated distances between the take-off and landing points in his study of platyrrhine monkeys, *Alouatta palliata* and *Cebus capucinus*; however, this was only for crossings that used leaping. Cannon and Leighton [32] used an alternative estimate of distance when comparing the gap crossing behaviour of gibbons *Hylobates agilis* and macaques *Macaca fascicularis*; they measured the distance between the terminal woody supports of gaps rather than the actual distance crossed by the primates. However, as this study involved a large range of body sizes it was not appropriate to measure the distance between the terminal branches because they were deflected according to the mass of the animal. We therefore followed Gebo’s [31] measurement of the distance from take-off to landing point; termed here the ‘actual gap crossed’ (AGC), but modified it slightly to account for the varied methods of gap crossing employed by orangutans. Thus, when the gap was crossed using mass deformation locomotion, the take-off point was estimated as the last location of the orangutan prior to support deformation (dashed tree outline in Fig 2a). For all other types of behaviour the take-off point was the last part of the take-off tree that the orangutan made contact with (Fig 2b). If this distance was estimated to be less than 0.5m the AGC was recorded as zero (Fig 2c). AGC was estimated to the nearest whole metre. In cases where an orangutan crossed a gap to get to a liana and then used the liana to get to the next tree, details of both crossings were recorded (Fig 2d).

### Statistical analysis

We examined which factors and combinations of factors influenced the size of gap an orangutan could cross by fitting a Generalised Linear Mixed Model using AGC as the response variable, and gap crossing behaviour and support type as predictors, with forearm length as a covariate. However, since the dependent variable AGC was composed of positive integers and had a large number of zeros (where the gap crossed was less than 0.5m), a Zero-Inflated Generalized Linear Mixed Model (ZIGLMM) was fitted with a Poisson distribution family and a log link, to accommodate the non-normal error structure. The modelling was performed using R 3.0.2 (R Development Core Team 2010), and the glmmADMB package (version 0.7.7). ZIGLMMs allow both fixed and random effects to be modelled, which was particularly important for the study because the data consisted of many observations collected from each individual. Individual ID was included as a random effect on the intercept. In order to provide biological context for the behaviours analysed in the ZIGLMM, we also performed chi-squared tests where possible on frequencies of the behaviours included in the model with age of the individual. Such tests were not possible for crossing behaviour because a high proportion of cells with an expected frequency of <5 would invalidate the test, but were possible for take-off support. The raw data is provided in S1 Dataset.

## Results

**Fig 3:**
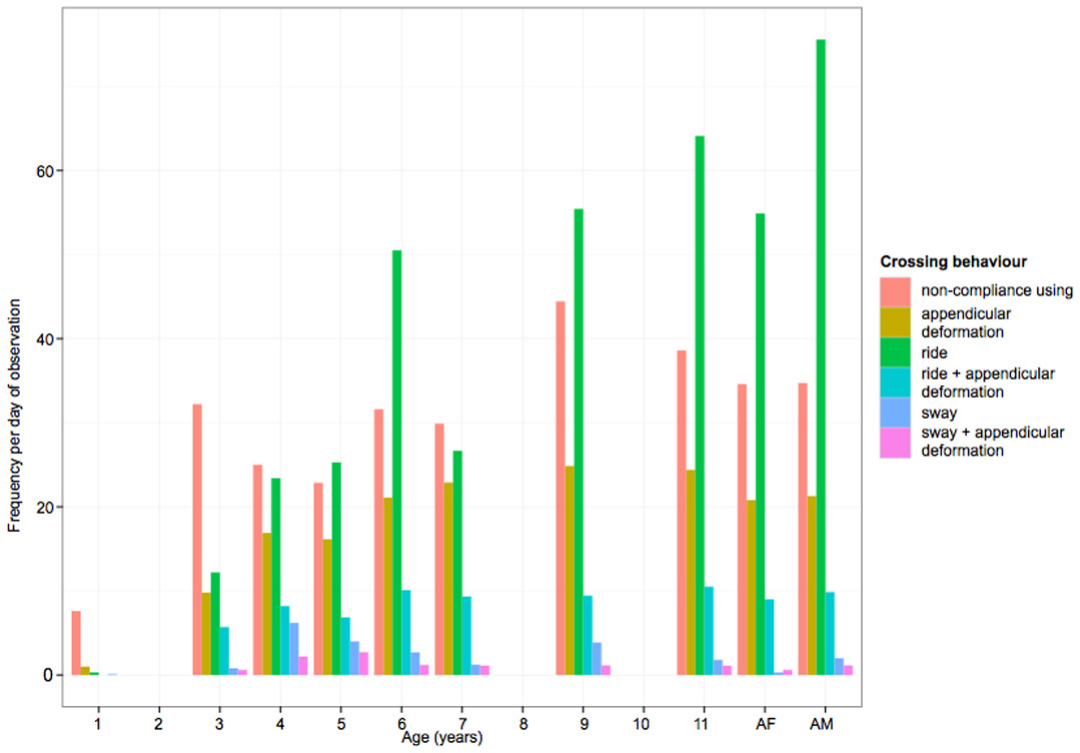
Frequency (per observation day) of gap crossing behaviours observed for different ages of orangutans. AF = adult females (Juni and Kerry), AM = adult males (Gismo and Preman). Crossing behaviour: NCU = non-compliance using crossing behaviour, APP = appendicular deformation, R = ride support, R+APP = ride support with appendicular deformation, S = sway support, S+APP = sway support with appendicular deformation. We were not able to sample individuals of 2, 8 and 10 years.

In total 8652 independent gap crossing events were recorded for the 12 orangutans studied. Overall we found that the youngest individual (1 year old) exhibited much less independent gap crossing behaviour than the other subjects, with 9 independent gap crossings observed per focal day compared to 61.3 gap crossings per focal day for the nearest individual in age, Kino (see Table 1). All other individuals exhibited at least 8.6 times as many independent gap crossings per focal day. Its gap crossing was dominated by NCU, although it was still exhibited infrequently compared to the other ages as it rarely crossed between trees without its mother (see Fig 3). There was a trend for the frequency of appendicular deformation (APP) to increase gradually with age until it reached its maximum value for the 9-year-old individual. Frequencies of ride combined with appendicular deformation (R+APP) similarly tended to increase until 6 years old and then plateau. Sway (S) and sway combined with appendicular deformation (S+APP) were generally low for all ages, but nevertheless peaked around the ages of 4 and 5 years. The largest difference in frequency of behaviour with age was seen for ride (R). The frequency of this behaviour increased with age and reached the highest frequency in the adult (>11-year-old) individuals. Thus, while the picture is somewhat complicated, there appears to be a greater overall frequency of gap crossing behaviour, and greater use of gap crossing methods that employ compliant supports, with age.

**Fig 4:**
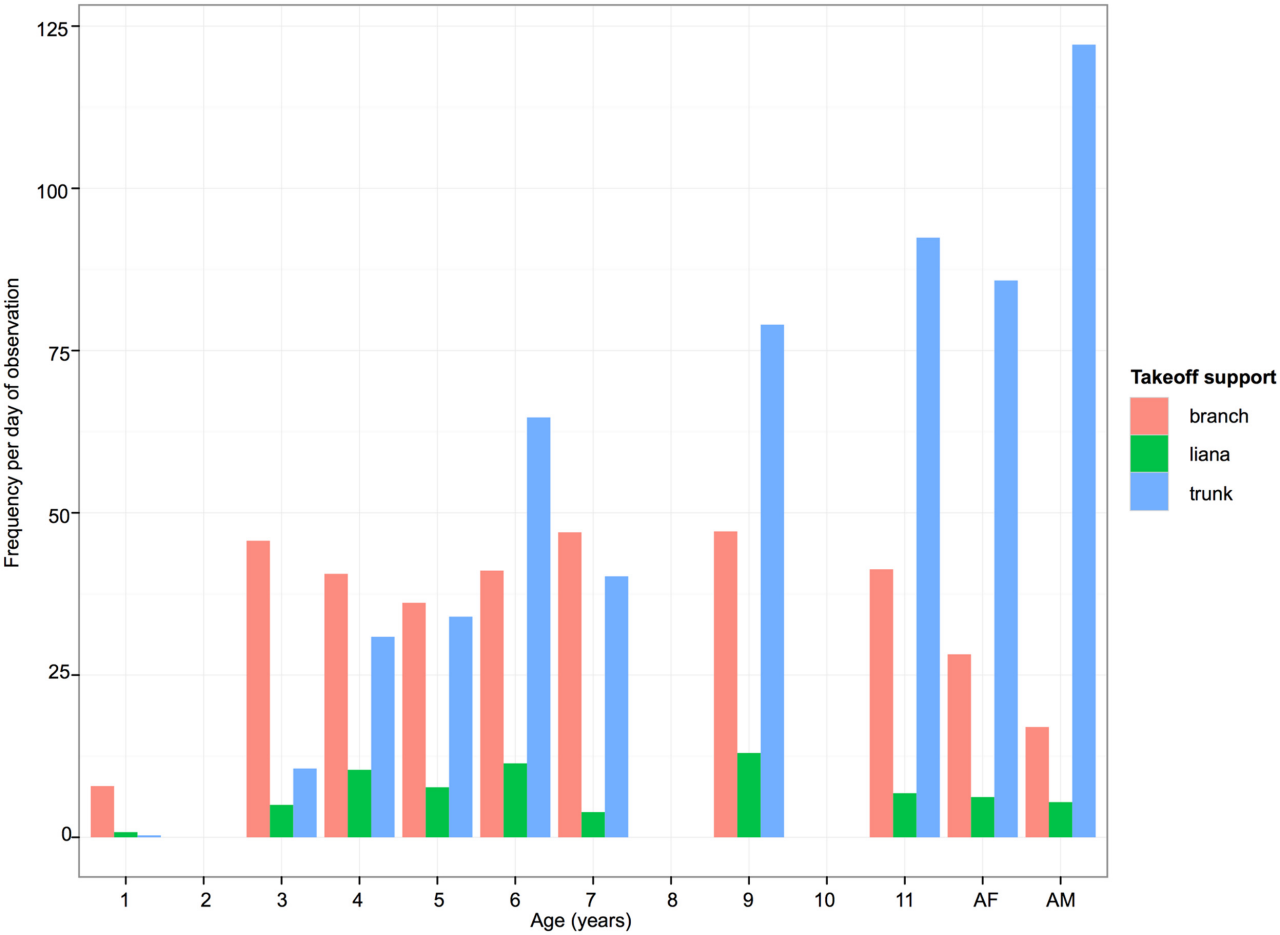
Frequency (per observation day) of three categories of take-off support, for different ages of orangutans. AF = adult females (Juni and Kerry), AM = adult males (Gismo and Preman).

Different age-classes of orangutans also appeared to differ in their choice of takeoff support while crossing gaps (see Fig 4 and Table 2). In this study use of lianas was at a relatively low frequency for individuals of all ages, but is lowest for the youngest and oldest individuals. The most striking trend is the increase in frequency of use of trunks with age, and the corresponding decrease in frequency of use of branches. A Pearson chi-squared test showed that frequency of use of take-off supports with age differed significantly from the expected distribution (Chi-sq = 144.76, df. = 18, p<0.0001). Inspection of the standardized cell residuals showed that individuals aged 1–4 years old used branches more frequently than expected and trunks less frequently than expected, while this pattern was reversed for adult females and males.

**Table 2:** Frequency (per observation day) of take-off support use with age category.

Age-sex	Branch	Liana	Trunk	Row total
1	7	0	0	9
	3.224	0.647	5.129	
	**2.604**	0.190	*-2*.*132*	
3	45	5	10	61
	21.961	4.406	34.933	
	**5.066**	0.283	*-4*.*117*	
4	40	10	30	81
	29.341	5.886	46.673	
	**2.079**	1.861	*-2*.*309*	
5	36	7	34	77
	27.893	5.596	44.369	
	1.562	-1.557	-1.557	
6	41	11	64	117
	41.987	8.423	66.790	
	-0.137	1.026	-0.256	
7	47	3	40	91
	32.641	6.548	51.922	
	**2.513**	-1.039	-1.624	
9	47	13	79	139
	49.848	10.000	79.294	
	-0.383	0.949	-0.033	
11	41	6	92	140
	50.335	10.098	80.068	
	-1.273	-1.038	1.378	
AF	28	6	85	120
	43.062	8.639	68.499	
	*-2*.*265*	-0.830	**2.090**	
AM	17	5	122	144
	51.793	10.390	82.388	
	*-4*.*835*	-1.539	**4.380**	
Column total	352	70	560	982

Each cell contains observed count, expected count, and standardized cell residual (SCR). SCRs ≥ +1.98 indicating a significantly greater frequency than expected are in bold, and SCRs ≤ -1.98 indicating a significantly lower frequency than expected are in italics.

We used a Zero-Inflated Generalised Linear Mixed Model (ZIGLMM) to test the effect on the actual gap crossed (AGC) of the fixed effects of crossing behaviour (6 levels) and take-off support type (3 levels), with forearm length (as a proxy for body size) as a covariate. Individual identity was included as a random effect on the intercept. The distribution family was specified as Poisson, and zero inflation was taken into account. After an iterative model fitting and criticism process, the best fitting model (minimizing the value of the AIC) included all the two-way interactions (the fully-saturated model including the three-way interaction could not be resolved using glmmADMB), as well as a random effect of individual on the intercept (1 | Individual). The model was as follows:

AGC ~ forearm + crossing behaviour + takeoff support + crossing behaviour * take-off support + forearm * crossing behaviour + forearm * take-off support + (1 | Individual).

The distribution of the residuals was checked and found to be normal with no obvious patterns of over or under-dispersion, non-homogeneity of variance, or other features that might invalidate the model. Significance of the interaction terms was determined by comparing nested models with and without the term of interest using an ANOVA (see Table 3). All three two-way interactions between the main effects were found to be highly significant (p<0.00001), thus estimates and corresponding significance levels are not given for the main effects.

**Table 3:** Deviance estimates, degrees of freedom and significance levels for interaction terms in ZIGLMM model, determined through an ANOVA test.

Interaction term	Deviance	df	*p* (Chi-sq distribution)
Forearm * take-off support	40.34	2	< 0.00001
Forearm * crossing behaviour	59.48	5	< 0.00001
Crossing behaviour * take-off support	231.32	10	< 0.00001

**Fig 5:**
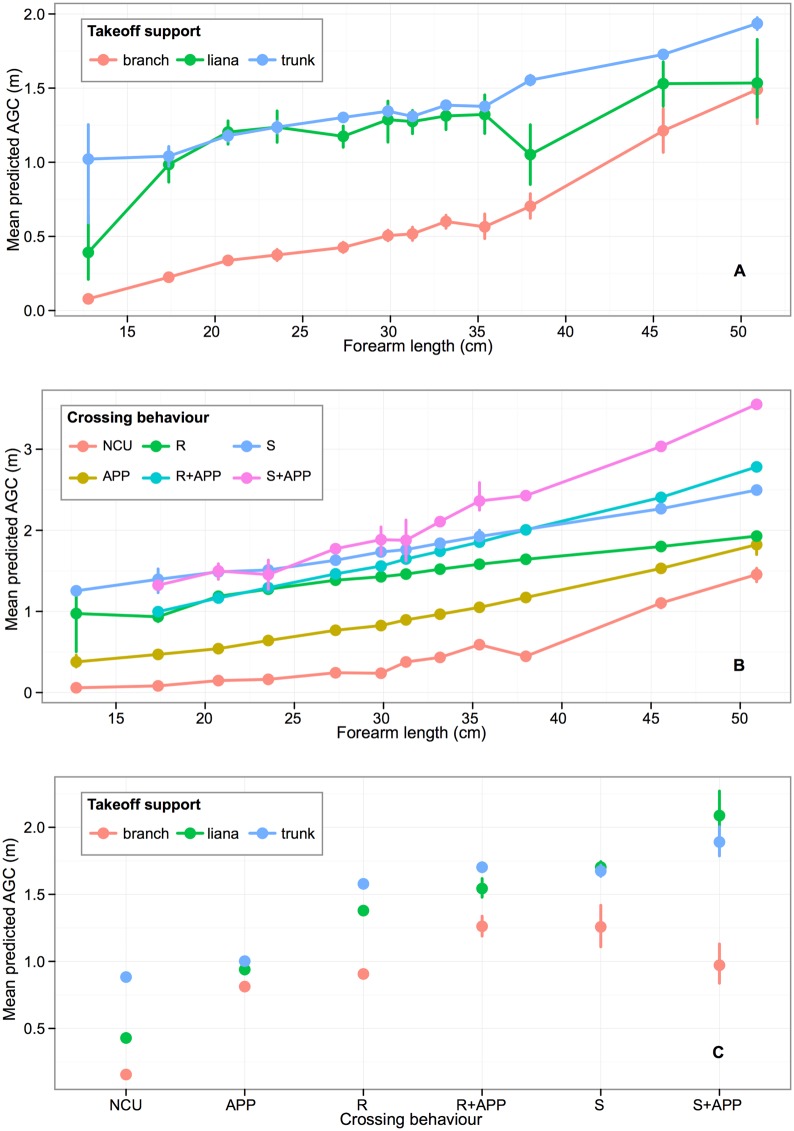
Mean predicted actual gap crossed (AGC) as a function of interactions between forearm length, crossing behaviour and takeoff support. Error bars are bootstrapped 95% confidence intervals on the mean. A) Interaction between forearm length and takeoff support. B) Interaction between forearm length and crossing behaviour. C) Interaction between takeoff support and crossing behaviour. Crossing behaviour: NCU = non-compliance using crossing behaviour, APP = appendicular deformation, R = ride support, R+APP = ride support with appendicular deformation, S = sway support, S+APP = sway support with appendicular deformation.

The gap an orangutan crossed when it moved between trees was therefore influenced by three interacting factors: the forearm length of the orangutan i.e. its body size, the type of crossing behaviour it employed, and the type of support it used in the take-off tree. The effect of forearm length was modified by both take-off support and crossing behaviour, and crossing behaviour was also modified by the take-off support used. Fig 5 illustrates the effect of the independent variables on the mean predicted AGC estimated by the model. There is a general trend for mean predicted AGC to increase with forearm length: longer forelimbs allow a larger gap to be crossed. However, for orangutans with a given forearm length, larger gaps were crossed when using lianas and particularly tree trunks than when using branches (Fig 5A), and when types of crossing behaviour involved more advanced use of support deformation (Fig 5B). Furthermore lianas and tree trunks facilitated a greater AGC for all forms of crossing behaviour, when compared to branches (Fig 5C). Similarly, the mean predicted AGC was lowest for gap crossing behaviours that did not employ the use of compliant supports (NCU), and increased for types of crossing behaviour employing compliance more extensively (e.g. ride, sway and combinations of those behaviours with appendicular deformation).

**Fig 6:**
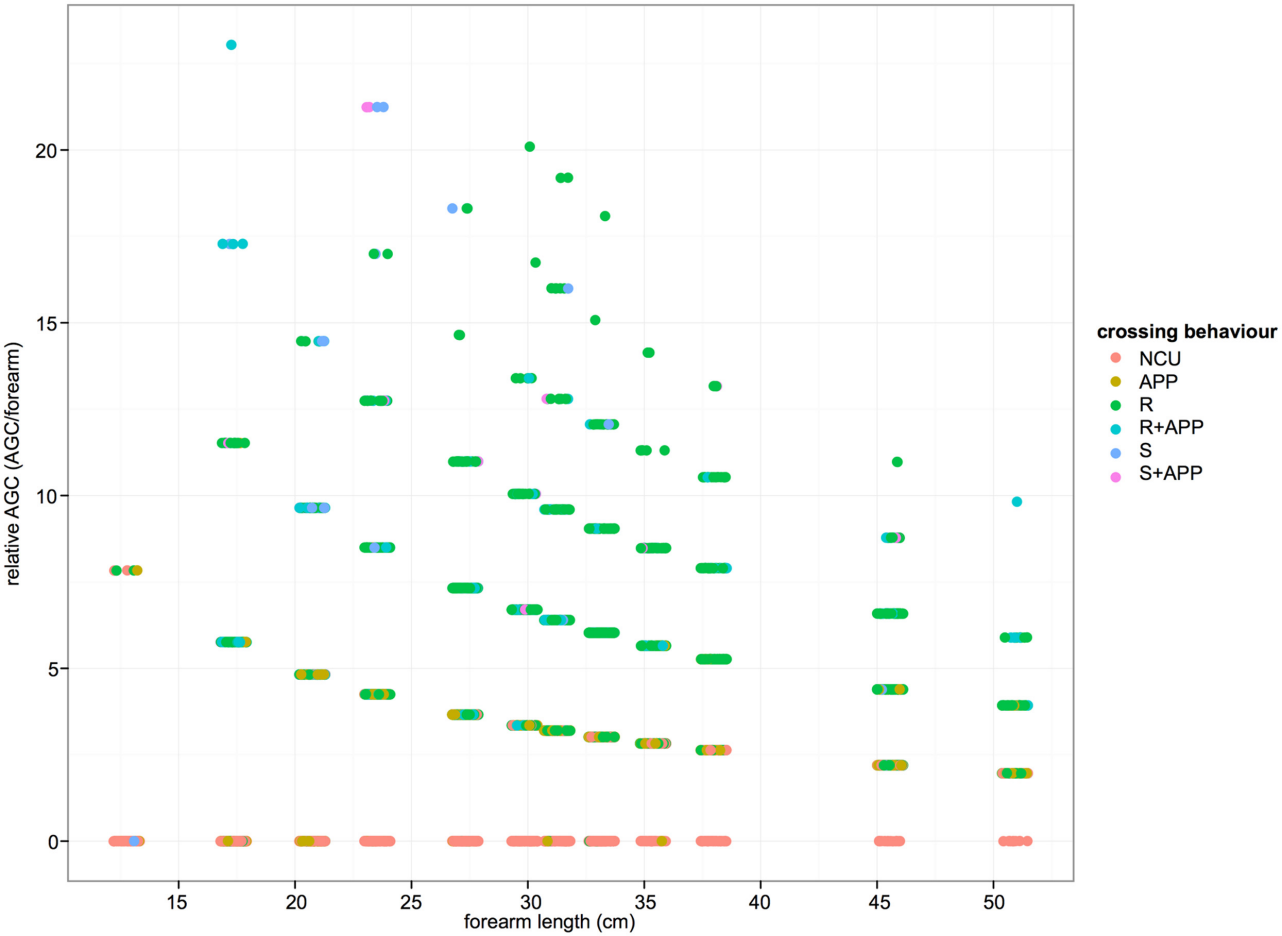
Relative AGC (AGC/forearm length) against forearm length, separated by type of crossing behaviour. Data points horizontally jittered to increase visibility.

In order to determine whether the size of gap that subjects crossed was strictly in proportion to their forearm length, relative AGC was calculated by dividing AGC by forearm length. Individuals with shorter forearm lengths showed a much wider range of relative AGC than those with longer forearms. For example, those with a forearm length of less than 35cm sometimes crossed gaps 15–22 times the length of their forearm, while those with forearms greater than 40cm long did not cross gaps more than 12 times their forearm length (see Fig 6). Furthermore, the instances of disproportionately large gaps crossed largely involved crossing behaviours where support deformation was involved.

## Discussion

Our results show that the frequencies of different types of gap crossing behaviour do change with age. The 1-year old exhibited substantially less independent gap crossing behaviour than the other age groups studied. Nevertheless, the results suggest that infants are capable of manipulating compliant vegetation to cross a gap by appendicular deformation already by the time they are 1 year old, many years before they attain nutritional independence [19, 27, 33]. This is in contrast to Bard’s [20] study which did not report independent gap crossing behaviour earlier than 2.5 years of age, but is in line with studies that have found that infants up to at least 2 yrs of age are carried by their mothers during most travel [19]. It is unfortunate that we were not able to obtain a sufficient dataset from 2-year old orangutans to better understand the nature of the transition from predominantly-carried to predominantly-independent travel.

The most complex forms of support manipulation (sway, and sway combined with appendicular deformation) both generally peaked in frequency between the ages of 4 and 5 years old. We suggest that this early peak reflects exploratory play behaviour where young orangutans practise more complex locomotor behaviours repeatedly to perfect their techniques. Since these behaviours appear to be the most cognitively demanding these results may suggest that the cognitive skills for complex forms of locomotion, such as where supports must be first oscillated away from the direction of travel, occur at around 4–5 years old. Interestingly 4–5 years old is also the age where the use of trunks for gap crossing increases sharply (Fig 4), which further suggests that this may be a period where young orangutans acquire the cognitive skills needed for manipulating more challenging (e.g. thicker) support types. The cognitive demands are not just imposed by the need to initiate the movement away from the direction of travel as in sway. An animal may enhance the effect of their mass on a compliant tree trunk by climbing further up, by climbing out along a branch or by changing the position of their centre of mass strategically while deforming a tree trunk, for example moving from on top of a branch to hanging below it once deformed to attain greater deflection. The need for such adjustments will vary substantially between trees, and also with the orangutan’s mass. It may be that it is these finer points of the manipulation of compliant supports that young orangutans master through play and frequent practice at around the age of 4–5 years old.

In contrast, frequencies of simple forms of support manipulation (appendicular deformation and ride) increased until 9 and 11 years old respectively where they reached a plateau, which suggests the requirements, and/or efficacy, of both of these may be exclusively associated with body size. These results complement studies that have quantified orangutan locomotion, both in dry lowland dipterocarp and swamp forest [3, 13–14], that have found striking consistency in the locomotor behaviours of all age-sex classes aged 7 years and above, and appears to suggest that adult patterns of locomotion are well developed by the age of completed weaning.

The gap an orangutan crossed when it moved between trees was found to be influenced by three interacting factors: orangutan forearm length as a proxy for body size, the type of crossing behaviour it used, and the type of support it used in the take-off tree. For a given forearm length orangutans were able to cross much larger gaps using lianas and tree trunks than branches, with tree trunks in particular magnifying the size of gap crossed in the largest two adults (the two males). Lianas often hang vertically from trees, which means that they can be oscillated without applying much force making them ideal supports for small orangutans to sway across gaps. In contrast, tree trunks tend to have larger diameters than branches and lianas and therefore require more force to deform them. However, when compared to branches, trunks tend to have a more vertical orientation that means that when they are deformed they allow the orangutan to cover greater horizontal distance than a more horizontal support. Additionally, in the degraded forest around Tuanan tree trunks with a suitable diameter to be readily deformed were far more numerous than lianas with a sufficient diameter to support the weight of a large orangutan, which may help young orangutans develop gap crossing skills earlier than those in more pristine forests. Lianas are extremely important in arboreal locomotion for a range of arboreal animals because they link tree crowns together bridging gaps and providing arboreal pathways. As this was the first study to investigate gap crossing behaviour of wild orangutans we cannot be sure how representative of other populations of orangutans in different habitats these data are. However our results appear to support Manduell et al’s [34] suggestion that tree trunks in degraded forest can be functionally equivalent to lianas in pristine forests for orangutans, which has important implications for determining crucial habitat requirements for sustaining orangutan populations and for understanding the energetic costs of orang-utan travel in different habitats.

Finally we found that smaller individuals crossed disproportionately large gaps relative to their size, which was facilitated by the use of support deformation. This probably reflects a combination of the more playful and exploratory nature of immature orangutans and the tendency for adult males and females to be more conservative in their locomotion, although for different reasons. Thorpe and Crompton [13] showed that parous females (both with and without clinging infants) selected larger supports for locomotion than juveniles, adult males and non parous females, and they are likely to have similarly attempted smaller gaps in this study. Adult males must be cautious by virtue of their size. However, we believe this study is the first to demonstrate an actual difference, since earlier studies have found remarkable similarity in the locomotion and support use of juveniles and adults of both sexes in dryland [3, 13], and peatswamp [14] forest.

The primary constraint of our study was the small number of individuals sampled in each age class due to orangutans having low densities and very long interbirth intervals. Nevertheless, our aim was to try to highlight large-scale patterns in the ontogeny of orangutan locomotion through a cross-sectional study, for which our sample size was adequate. Overall, our results suggest that immature orangutans acquire and practice all the locomotor skills necessary to navigate their arboreal habitat before the age of weaning. Similarly, nest building, diet and foraging skills (including tool use) are also largely acquired before weaning [18–19, 35–36]. Post-weaning, the frequency with which they use the various techniques still changes, with increasing body size, but gap crossing patterns seems to stabilize before the age of first reproduction.

## Conclusions

This study has found evidence that the gap crossing behaviour of orangutans varies with both physical and cognitive development. Our results supports our hypothesis that the exploitation of compliance during gap crossing will increase as body size increases, although we found that even 1-year old infants were capable of manipulating compliant vegetation to cross a gap by appendicular deformation. Although we upheld our prediction that the skills required to cross a gap using mass deformation will develop later than appendicular deformation skills due to their cognitive and physical demands, the more complex forms of support manipulation (sway, and sway and appendicular deformation combined) peaked in frequency between the ages of 4 and 5 years old. We suggest this relates to locomotor play behaviour, but it may also represent a period where young orangutans acquire the cognitive and physical skills needed for manipulating more challenging support types. Overall adult patterns of locomotion appear to be broadly present by about 6–7 years old, thus around the time of weaning despite these individuals still being substantially smaller than adults. Finally, although our results supported the hypothesis that the size of the gap crossed will increase as body size increases, we found that smaller individuals crossed larger gaps relative to their size than larger individuals. We suggest that this relates to exploratory youngsters testing their abilities and developing new skills through play and to conservative adults minimising the risk of falls.

## Supporting Information

S1 Dataset Chappell et al dataset. (XLSX)
